# Gene-Silencing-Induced Changes in Carbohydrate Conformation in Relation to Bioenergy Value and Carbohydrate Subfractions in Modeled Plant (*Medicago sativa*) with Down-Regulation of *HB12* and *TT8* Transcription Factors

**DOI:** 10.3390/ijms17050720

**Published:** 2016-05-13

**Authors:** Xinxin Li, Abdelali Hannoufa, Yonggen Zhang, Peiqiang Yu

**Affiliations:** 1 College of Animal Science and Technology, Northeast Agricultural University, Harbin 150030, China; youlixinxin@163.com; 2 Agriculture and Agri-Food Canada, 1391 Sandford Street, London, ON N5V 4T3, Canada; abdelali.hannoufa@agr.gc.ca; 3 College of Agriculture and Bioresources, University of Saskatchewan, 51 Campus Drive, Saskatoon, SK S7N 5A8, Canada

**Keywords:** gene-silencing induced changes, molecular structure, biofunctions, nutrient bioavailability, alfalfa forage, functional groups of carbohydrates

## Abstract

Gene silencing with RNA interference (RNAi) technology may be capable of modifying internal structure at a molecular level. This structural modification could affect biofunctions in terms of biodegradation, biochemical metabolism, and bioactive compound availability. The objectives of this study were to (1) Detect gene silencing-induced changes in carbohydrate molecular structure in an alfalfa forage (*Medicago sativa* spp. *sativa:* alfalfa) with down-regulation of genes that encode transcription factors *TT8* and *HB12*; (2) Determine gene silencing-induced changes in nutrient bioutilization and bioavailability in the alfalfa forage (*Medicago sativa*); and (3) Quantify the correlation between gene silencing-induced molecular structure changes and the nutrient bioutilization and bioavailability in animals of ruminants. The experimental treatments included: T1 = Non-transgenic and no-gene silenced alfalfa forage (code “NT”); T2 = *HB12*-RNAi forage with *HB12* gene down regulation (code “*HB12*”); T3 = *TT8*-RNAi forage with *TT8* gene down regulation (code “*TT8*”). The *HB12* and *TT8* gene silencing-induced molecular structure changes were determined by non-invasive and non-destructive advanced molecular spectroscopy in a middle infrared radiation region that focused on structural, non-structural and total carbohydrate compounds. The nutrient bioutilization and bioavailability of the modified forage were determined using NRC-2001 system in terms of total digestive nutrient (TDN), truly digestible fiber (tdNDF), non-fiber carbohydrate (tdNDF), fatty acid (tdFA), crude protein (tdCP) and bioenergy profiles (digestible energy, metabolizable energy, net energy) for ruminants. The carbohydrate subfractions were evaluated using the updated CNCPS 6.0 system. The results showed that gene silencing significantly affected tdNFC (42.3 (NT) *vs.* 38.7 (*HB12*) *vs.* 37.4% Dry Matter (*TT8*); *p =* 0.016) and tdCP (20.8 (NT) *vs.* 19.4 (*HB12*) *vs.* 22.3% DM (*TT8*); *p =* 0.009). The gene-silencing also affected carbohydrate CA4 (7.4 (NT) *vs.* 4.2 (*HB12*) and 4.4% carbohydrate (CHO) (*TT8*), *p =* 0.063) and CB1 fractions (5.3 (NT) *vs.* 2.0 (*HB12*) and 2.6% CHO (*TT8*), *p =* 0.006). The correlation study showed that the structural CHO functional group peak area intensity at *ca.* 1315 cm^−1^ was significantly correlated to the TDN_1x_ (*r* = −0.83, *p =* 0.042) and the tdNFC (*r =* −0.83, *p =* 0.042), the structural CHO functional group height intensity at *ca.* 1370 cm^−1^ was significantly correlated to the tdNDF (*r* = −0.87, *p =* 0.025). The A_Non-stCHO to A_StCHO ratio and A_Non-stCHO to A_CHO ratio were significantly correlated to the tdFA (*r* = 0.83–0.91, *p* < 0.05). As to carbohydrate fractions, both CA4 and CB1 correlated with carbohydrate spectral intensity of the H_1415 and the H_1315 (*p =* 0.039; *p =* 0.059, respectively), CB3 tended to correlate with the H_1150, H_1100 and H_1025 (*p <* 0.10). In conclusion, RNAi-mediated silencing of *HB12* and *TT8* modified not only inherent CHO molecular structure but also the biofunctions. The CHO molecular structure changes induced by RNAi gene silencing were associated with biofunctions in terms of the carbohydrate subfractions and nutrient digestion.

## 1. Introduction

Gene silencing through RNAi technology is capable of modifying internal structure at a molecular level. The structural modification could affect biofunctions in terms of biodegradation, biochemical metabolism, and bioactive compound availability [1,2,3,4]. Jonker *et al.* [1] reported that insertion of the *Lc* gene into three winter-hardy alfalfa varieties in western Canada induced production of proanthocyanidin (PA) and anthocyanidin (AC) compounds, which are not produced in non-transgenic alfalfa plants. The PA and AC compounds are capable of bonding with highly soluble forage protein in the rumen, preventing soluble protein from being degraded in the rumen and shifting protein from the rumen to the small intestine to be digested by internal enzymes released from the small intestine. This genetic modification was shown to reduce incidences of bloating and digestive disorders, thus resulting in improvement of nutrient availability in ruminants [2,3,4]. 

Yu *et al.* [5] reported that not only was nutrient availability improved by *Lc* gene transformation [1,2,3,4], but also protein molecular structure profiles [5] were modified by *Lc* transformation in terms of protein amide I, amide II, amide I to amide II ratio, protein structural alpha-helix, beta-sheet, random coil and bête-turns and their ratios [5]. 

Recently, two novel RNAi alfalfa genotypes were developed by down-regulating the expression of the alfalfa *TT8* and *HB12* genes [6]. These two genes are expected to modify the lignin biosynthesis pathway resulting in reducing the forage lignin level and affecting lignin structural conformation [6]. Genetically, manipulation of plant cell wall properties has the potential to improve bioenergy production, as the lignin present in the plant secondary cell walls negatively correlates with sugar release. Thus, we could successfully modify lignocellulosic materials to improve saccharification in alfalfa [7]. It is also expected that if lignin biosynthesis is modified, biosynthesis of PA and CA compounds will also be affected in a favorable way to increase levels of PA and AC compounds, which may result in a net improvement of nutrient utilization and availability. However, to date, no study has been carried out to study the association of molecular structure changes with nutrient availability in the *HB12* and *TT8* gene-modified alfalfa forage.

The objectives of this study were to (1) Detect gene silencing-induced changes in carbohydrate molecular structure in modified alfalfa forage (*Medicago Sativa*) with down-regulation of *TT8* and *HB12* genes; (2) Determine gene silencing-induced changes in nutrient bioutilization and bioavailability in the modified alfalfa forage; and (3) Quantify the relationship between gene silencing-induced molecular structure changes and the nutrient bioutilization and bioavailability in ruminants.

The hypothesis of this study was that silencing *HB12* and *TT8* genes in alfalfa would induce molecular structure changes, which would result in biological changes in terms of carbohydrate subfractions and nutrient availability. 

## 2. Results and Discussion

### 2.1. Changes in Nutrient Bioutlization and Bioavailability in Alfalfa with Down-Regulated TT8 and HB12 Genes

Truly digestible nutrients and bioenergy profiles, evaluated using NRC-2001 [8] and NRC-1996 [9], that were affected by gene-silencing in the alfalfa forage (*Medicago sativa:* Alfalfa) are presented in Table 1. Both *HB12*-RNAi and *TT8*-RNAi alfalfa were lower in tdNFC content than non-transgenic alfalfa population (38.7 and 37.4 *vs.* 42.3% DM; *p* < 0.05). *TT8*-RNAi alfalfa had highest tdCPc value compared with *HB12*-RNAi alfalfa and non-transgenic alfalfa (22.3 *vs.* 19.4 and 20.8% DM; *p* < 0.05). There were no differences in tdFA and tdNDF among the NT, *HB12* and *TT8* groups with an average of 0.63% DM for tdFA and 14.7% DM for tdNDF. The TDN_1x_ and bioenergy values (DE_1x_, NE_m_, NE_g_, DE_3x_, ME_3x_ and NE_LP_) in HB12-RNAi and TT8-RNAi alfalfa tended to be lower than in non-transgenic alfalfa population (*p* < 0.10) with TDN1x = 71.9 *vs.* 68.2% DM; DE_1x_ 3.31 *vs.* 3.15; ME_3x_ 2.62 *vs.* 2.45; NE_m_ 1.79 *vs.* 1.69; NE_g_ 1.17 *vs.* 1.07 and NE_L_ 1.65 *vs.* 1.54 Mcal/Kg DM for non-transgenic group and gene silencing groups, respectively.

Little information is available in published literature on the effect of genetic modifications on forage bioenergy value, truly digestible nutrient and total digestible nutrient. The only information that could be found in literature is from Jonker *et al.* [1,2,3,4] who found that transformation of alfalfa with *Lc* gene increased PA and AC compounds and thus improved nutrient availability in dairy cattle. However, Jonker *et al.* [1,2,3,4] did not determine the specific bioenergy values and TDN and truly digestible nutrients (tdNDF, tdCP, tdFA and tdNFC) for *Lc*-transgenic alfalfa. No reports have been found on the effect of gene silencing on bioenergy value in forage. Our results indicate that silencing of *HB12* and *TT8* genes in alfalfa affected not only the biochemical biosynthesis pathway but also the bioenergy profile and truly digestible nutrients for ruminants. 

### 2.2. Effects of TT8 and HB12 Silencing on Carbohydrate Sub-Fractions in Alfalfa Forage

Previous studies showed that alfalfa transformation with *Lc* gene changed both protein and carbohydrate subfractions in winter-hardy alfalfa populations [1,2]. These subfraction changes also resulted in alterations in rumen degradable and undegradable fractions. However, no study has been reported on the effect of gene-silencing on carbohydrate fractions. 

In this study, carbohydrate pools were partitioned into eight sub-fractions (CA1, CA2, CA3, CA4, CB1, CB2, CB3, CC) according to the updated CNCPS system (version 6.0), [10,11]. These fractions are a biological reflection of rumen fermentation characteristics in dairy cows [10,11]. According to the AMTS database 2010, professional [12], freeze dried alfalfa rarely contains organic acids. This was confirmed in these new alfalfa populations (Table 2). Table 2 also shows the effects of the *TT8* and *HB12* silencing on carbohydrate subfractions in alfalfa forage. The non-transgenic alfalfa population tended towards higher CA4 (7.37 in NT *vs.* 4.18 in *HB12* and *TT8* in 4.41% CHO; *p =* 0.063) than the *HB12*- and *TT8*-RNAi alfalfa populations (*p* < 0.10). These transgenic alfalfa populations were also 50% lower in the starch-containing sub-fraction (CB1, *p* < 0.05). The gene-silencing did not affect carbohydrate CB2 soluble fiber fraction which was intermediately degradable carbohydrate in the rumen (49.4 in NT *vs.* 49.8 in *HB12* and 49.4 in *TT8*, *p* > 0.10) and carbohydrate CB3 fractions (available neutral detergent fiber) which were slowly degradable carbohydrate in the rumen (29.9 in NT *vs.* 29.6 in *HB12* and 31.2 in *TT8*, *p* > 0.10). Our results clearly indicated that the *HB12-* and *TT8*-RNAi gene silencing only affects fast degradable carbohydrate fractions but not slowly and intermediately degradable carbohydrate fractions in the rumen.

### 2.3. Multivariate Analysis of Carbohydrate Molecular Spectral Profiles that Are Affected by HB12 and TT8 Silencing

**Figure 1:**
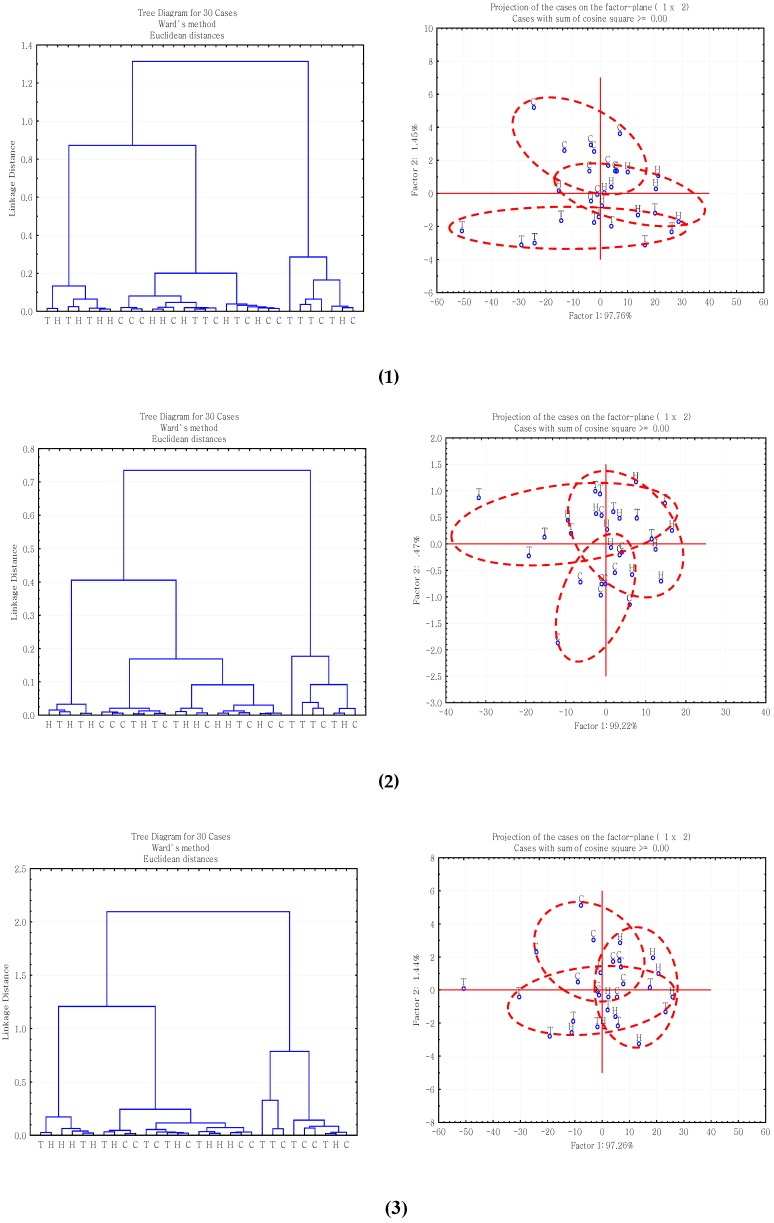

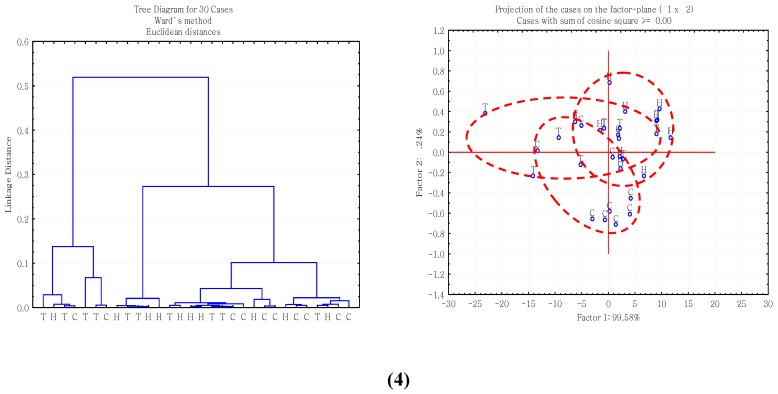
Hierarchical cluster analysis (CLA) and principal component analysis (PCA) of *HB12* RNAi alfalfa (H), *TT8* RNAi alfalfa (T) and nontransgenic alfalfa (C) for spectral region: (1) structural carbohydrate fingerprint region: *ca*. 1485–1188 cm^−1^; (2) cellulosic compound fingerprint region: *ca*. 1294–1188 cm^−1^; (3) total carbohydrate fingerprint region: *ca.* 1190–930 cm^−1^; (4) non-structural carbohydrate fingerprint region: *ca*. 931–875 cm^−1^. Cluster methods (Ward’s algorithm) and distance measure (Eculidean distances) were used in CLA analysis. Scatter plots of two principal components (PC1 vs. PC2) in PCA analysis.

The first multivariate analysis is agglomerative hierarchical cluster analysis (CLA) based on Ward’s algorithm method, which has been successfully used for discriminating functional groups existing in inherent molecular structure of feeds [13,14]. The second multivariate analysis method is principal component analysis (PCA), which is widely used for investigating major sources of variation in feed spectra [15,16]. Figure 1 displayed PCA and CLA analysis results of carbohydrate related fingerprint regions: (1) structural carbohydrate fingerprint region: *ca.* 1485–1188 cm^−1^; (2) cellulosic compound fingerprint region: *ca.* 1294–1188 cm^−1^; (3) total carbohydrate fingerprint region: *ca.* 1190–930 cm^−1^; and (4) non-structural carbohydrate fingerprint region: *ca.* 931–875 cm^−1^. 

The cluster classes of these four regions could not be distinguished from each other, which implied that the inherent carbohydrate-related molecular structures are highly related to each other between the non-transgenic and the two RNAi gene silenced alfalfa populations. In four PCA figures, the first principal component explained 97.76%, 99.22%, 97.26% and 99.58% of the variation, respectively. The principal components (PCs) of the three different genotypes alfalfa populations overlapped, which supported the result of CLA analysis that no separate groups could be obtained among the RNAi alfalfa and non-transgenic alfalfa populations in these four carbohydrate-related spectral regions using the PCA method.

Both CLA and PCA analyses indicated that the inherent carbohydrate-related molecular structures of the RNAi silenced and non-transgenic alfalfa populations were highly related to each other. Similar CLA analysis results had been found in a previous study of molecular structure in three winter-hardy *Lc*-transgenic alfalfa populations with advanced synchrotron radiation-based IR microspectroscopy [5].

### 2.4. Relationship between Gene-Silencing-Induced Molecular Structure Changes and Nutrient Bioutilization and Bioavailability and Bioenergy of Alfalfa Forage

Molecular structure spectral parameters are sensitive to nutrient profiles and availability in ruminants [17,18]. The correlation between carbohydrate molecular spectral characteristics and truly digestible nutrients and bioenergy is presented in Table 3. The spectral intensity of A_StCHO was negatively correlated with tdNDF (*r* = −0.81, *p* < 0.05). Similar negative correlation could be found in the study of bioethanol co-product DDGS [19]. The biological meaning is that when spectral intensity of A_StCHO increases, truly digestible neutral detergent fiber will decrease. The spectral intensity of A_1315 had a negative correlation with TDN_1x_ (*r* = −0.83, *p* < 0.05) and tdNFC (*r* = −0.83, *p* < 0.05). This means that A_1315 could be a good predictor of total digestible nutrient and non-fiber carbohydrate. Both H_1244 (*r* = −0.85, *p* < 0.05) and A_CELC (*r* = −0.83, *p* < 0.05) were negatively associated with tdFA. CELC peak area and tdFA had a negative correlation (*r* = −0.71, *p =* 0.05) in a previous report [19]. A_Non-stCHO to A_CHO ratio (*r* = 0.83, *p* < 0.05), A_Non-stCHO/A_StCHO ratio (*r* = 0.91, *p* < 0.05) and A_CELC/A_CHO ratio (*r* = −0.94, *p* < 0.05) were also correlated with the concentration of tdFA. Total CHO-related spectral region tended to negatively related to tdNDF content (*p* < 0.05). No correlation was found between the carbohydrate structure spectral profiles and bioenergy profiles (*p* > 0.10). There are no other studies in the literature on the relationship between carbohydrate molecular structure spectral profile and nutrient availability and bioenergy profiles, and thus no comparison could be made. Our results showed that molecular structure spectral profiles are highly associated with total and truly digestible nutrient but not bioenergy values.

### 2.5. Relationship between Gene Silencing-Induced Molecular Structure Changes and Carbohydrate SubFractions in Alfalfa Forage Populations

Carbohydrate subfractions included fast, intermediate and slowly degradable carbohydrate fractions. These fractions link to nutrient availability in the rumen and in the small intestine in ruminants. Table 4 presents the relationship between gene silencing-induced molecular structure changes and carbohydrate subfractions in the alfalfa forage populations. The results showed that the peak height intensity of H_1415 had a positive correlation with CA4 fraction (*r* = 0.83, *p <* 0.05) and CB1 fraction (*r* = 0.83, *p* < 0.05), respectively. This result indicated that the spectral parameter of H_1415 peak intensity was affected highly by fast degradable carbohydrate fraction in the rumen. The spectral intensity of H_1370 was negatively associated with CB3 fraction (*r* = −0.81, *p =* 0.05), and tended to positively associated with CA4 fraction (*r* = −0.75, *p* < 0.10) and CB1 fraction (*r* = −0.75, *p* < 0.10). These results indicated that H_1370 spectral intensity was affected by both fast and slowly degradable carbohydrate fractions. Peak area intensity of A_1315 was positively correlated with CC fraction (*r* = 0.83, *p* < 0.05). It means that the higher the A_1315 intensity of CHO, the higher the undegradable carbohydrate fraction. Therefore, A_1315 could be a good predictor of indigestible fractions in the alfalfa forage. The A_CHO, H_1150, H_1100 and H_1025 all tended to have similar positive correlation with both CA4 fraction and CB1 fraction (*p* < 0.10). In the previous study on hulless barley [20], CA was negatively associated with CHO peak area (*r* = −0.10, *p* < 0.05), whereas, CB1 had a positive correlation with CHO peak area (*r* = 0.92, *p* < 0.05).

### 2.6. Predictions for Gene-Silencing-Induced Molecular Structure Changes and Nutrient Availability of Alfalfa Forage

The multiple regression analysis used to select the best spectral parameters to predict carbohydrate nutrient supply and availability in the gene-silenced and non-transgenic alfalfa populations is shown in Table 5. The results clearly showed that carbohydrate utilization for dairy cattle was highly related to carbohydrate molecular structure. A_StCHO/A_CHO ratio was the most important parameter that could be used to predict carbohydrate CA4 fraction in the alfalfa forage. The spectral parameter of H_1415 was an important index/predictor of the value of carbohydrate CB1 fraction. A_non-STCHO was a better predictor of tdNDF.

## 3. Experimental Section

### 3.1. HB12-RNAi, TT8-RNAi and Non-Transgenic Alfalfa Population Material

Alfalfa (*Medicago sativa*) clone N4.4.2 [21] was used as the wild type control, and as the recipient for transformation with *HB12* and *TT8* RNAi constructs. The alfalfa clone was obtained from Daniel Brown (Agriculture and Agri-Food Canada, AAFC, London, ON, Canada). All alfalfa plants were grown under greenhouse conditions at 21–23 °C, 16 h light per day with halogen lights having been applied after 18:00 h. Light intensity of 380–450 W/m^2^ (~500 W/m^2^ at high noon time) and a relative humidity of 70% were maintained throughout the growth period.

Harvests of individual plants were conducted at early-to-mid vegetative stage. Plants were stored in bags. Each bag represented one cut of one plant grown in a spot in the greenhouse. The *HB12* RNAi genotype had 11 bags in total, which were divided into two replicated samples. The *TT8* RNAi genotype had five bags, which were also divided into two replication samples. Harvests from each genotype were freeze-dried individually for each plant and ground through a 1-mm screen (Retsch ZM-1, Brinkmann Instruments Ltd., Mississauga, ON, Canada) at the Department of Animal and Poultry Science, University of Saskatchewan. Two replicate samples of each genotype population were drawn from individual plants (combining different individual plants within each genotype). Alfalfa populations were named *TT8* RNAi alfalfa (*n* = 2), *HB12* RNAi alfalfa (*n* = 2), and control alfalfa (*n* = 2).

#### Generating RNAi Constructs and Transformation of Alfalfa

Extraction of RNA, making of HB12 and TT8 RNAi constructs, and transformation of alfalfa were conducted as described in Li *et al.*, 2015 [6].

### 3.2. Advanced Non-Invasive Molecular Spectroscopy-FT/IR

**Figure 2:**
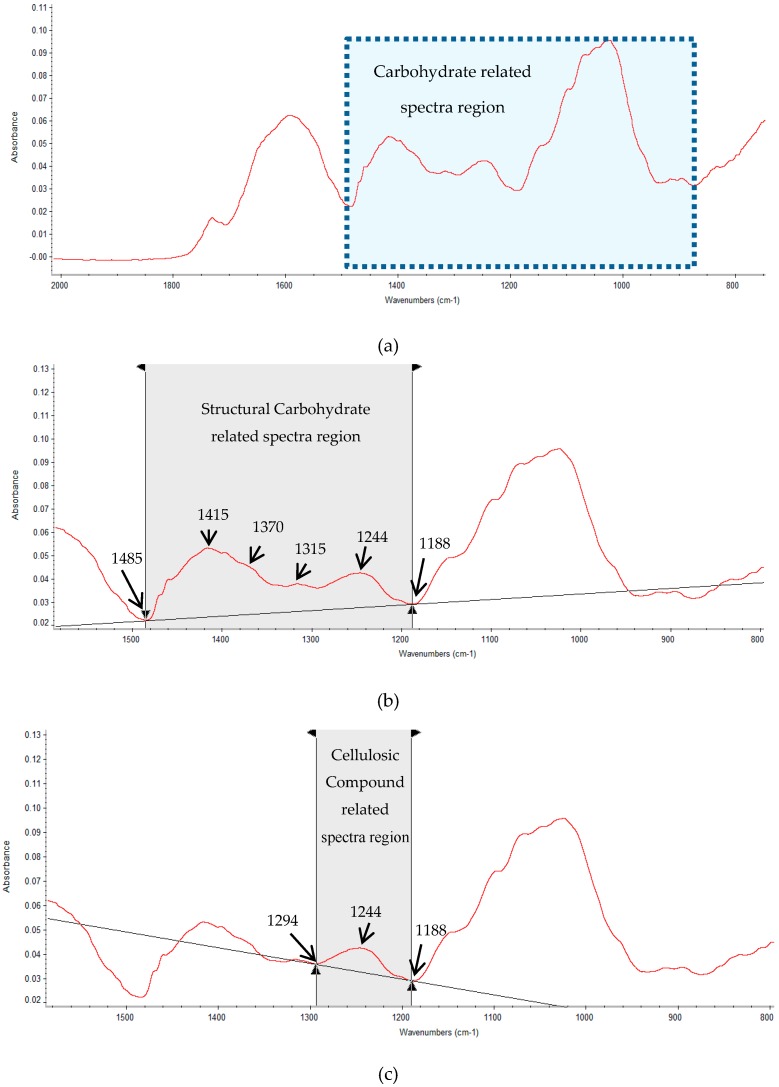

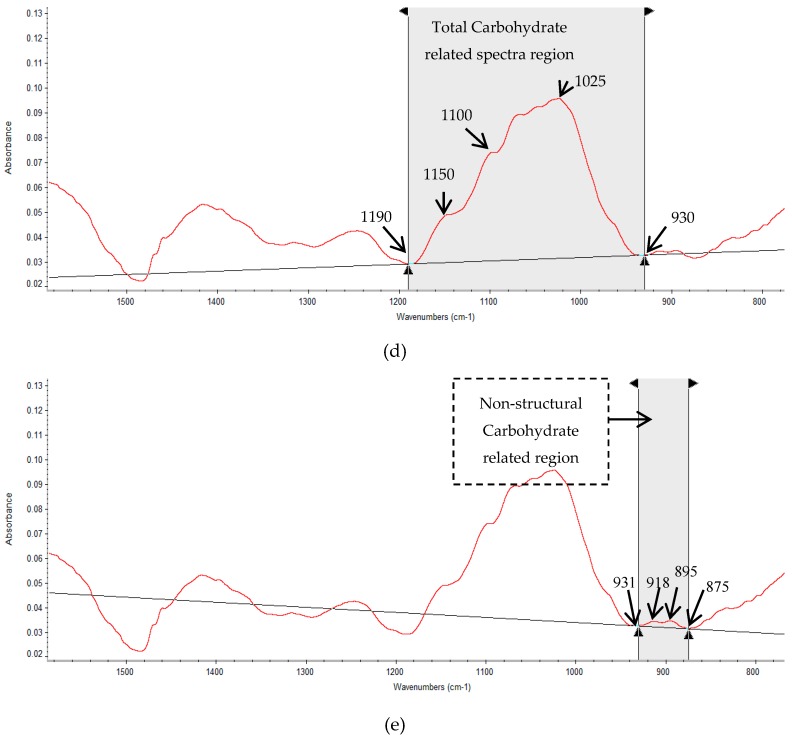
Typical ATR/FTIR molecular spectral for alfalfa: (**a**) whole mid-IR spectrum region (*ca.* 4000–800 cm^−1^); (**b**) structural carbohydrate related spectrum region (*ca*. 1485–1188 cm^−1^); (**c**) cellulosic compound related spectrum region (*ca*. 1294–1188 cm^−1^); (**d**) total carbohydrate related spectrum region (*ca*. 1190–930 cm^−1^); (**e**) non-structural carbohydrate related spectrum region (*ca*. 931–875 cm^−1^).

Fourier-transformed infrared-vibration (FT/IR) spectroscopy experiments were carried out at APS molecular spectroscopy lab, the University of Saskatchewan, Saskatoon, SK, Canada to detect carbohydrate-related molecular structure spectral features. The alfalfa forage samples were freeze-dried and ground through a 0.50 mm screen with Retsch ZM-1 (Brinkmann Instruments Ltd.). The FT/IR spectral data were obtained from the mid-IR region (*ca.* 4000–800 cm^−1^) at a resolution of 4 cm^−1^ and 128 co-added scans by JASCO SpectraManager II software, using JASCO FT/IR-4200 (JASCO Corporation, Tokyo, Japan). The IR molecular spectroscopy instrument equipped with a ceramic infrared light source and a deuterated l-alanine doped triglycine sulfate detector (JASCO Corp., Tokyo, Japan), employing a MIRacle ATR accessory module, as well as a ZnSe crystal and pressure clamp (Pike Technologies, Madison, WI, USA). Each sample was analyzed in five times. Typical spectra bands are presented in Figure 2.

The collected spectra were processed using OMNIC 7.3 software (Spectra Tech, Madison, WI, USA). In accordance with other published studies [16,17,22,23], the well-known molecular spectral parameters for carbohydrate-related functional groups in this study were included: (1) a broad structural CHO peak area (A_StCHO, region and baseline: *ca.* 1485–1188 cm^−1^) featuring three major peaks at *ca.* 1415, 1370, and 1315 cm^−1^; (2) a cellulosic compound peak area (A_CELC, region and baseline: *ca.* 1294–1188 cm^−1^) centered at *ca.* 1244 cm^−1^; (3) a total CHO peak area (A_CHO, region and baseline: *ca.* 1190–930 cm^−1^) featuring three major peaks at *ca.* 1150, 1100, and 1025 cm^−1^; and (4) a non-structural CHO peak area (A_Non-stCHO, region and baseline: *ca.* 931–875 cm^−1^) with two peaks at *ca.* 918 and 895 cm^−1^ (Figure 2). The ratio of each particular peak intensity was based on individual peak area values.

### 3.3. Multivariate Molecular Spectral Analyses of CHO Spectral Data Collected from Alfalfa Forage (Medicago sativa) with Down-Regulation of TT8 and HB12 Genes

Agglomerative hierarchical cluster analysis (CLA) and principal component analysis (PCA) were applied to the analysis of carbohydrate-related molecular spectral data using Statistica software version 8.0 (StatSoft Inc., Tulsa, OK, USA). Multivariate analysis of the carbohydrate-related fingerprint regions, including structural CHO (*ca.* 1485–1188 cm^−1^), cellulosic compound (*ca.* 1294–1188 cm^−1^), total CHO (*ca.* 1190–930 cm^−1^), and non-structural CHO (*ca.* 931–875 cm^−1^) were used to discriminate inherent differences in cellulosic compound, and to clarify variation within the spectral regions among the three populations (NT *vs.* *HB12 vs.* *TT8*). For each alfalfa sample, we analyzed five times, and got five subsamples.

### 3.4. Evaluation of Nutrient BioUtilization and Bioavailability and BioEnergy Profiles in Alfalfa Forage (Medicago sativa) with Down-Regulation of TT8 and HB12 Genes

The processing and chemical analysis methods of alfalfa sample were described in Li *et al.*, 2015 [6]. Evaluation of bioutilization and bioavailability included: Digestible energy (DE_1x_), truly digestible fatty acid (tdFA), truly digestible NDF (tdNDF), truly digestible NFC (tdNFC), truly digestible CP (tdCP) and total digestible nutrient at a maintenance level (TDN_1x_) which were evaluated using NRC-2001 [8] with a summative approach [24]. The bioenergy included: metabolizable energy (ME_3x_), digestible energy (DE_3x_), net energy for lactation (NE_LP_), net energy for maintenance (NE_m_) and net energy for growth (NE_g_) evaluated using NRC-2001 and NRC-1996 [8,9,24].

### 3.5. Partitioning Carbohydrate Subfractions in Alfalfa Forage (Medicago sativa) with Down-Regulation of TT8 and HB12 Genes

According to the updated Cornell Net Carbohydrate and Protein System (CNCPS, Version 6.0) [10,25], carbohydrate in the alfalfa samples was partitioned into eight sub-fractions: CA1 (volatile fatty acids), CA2 (lactate acid), CA3 (other organic acid), CA4 (sugar), CB1 (starch), CB2 (soluble fiber), CB3 (available NDF), and CC (indigestible fiber).

### 3.6. Statistical Analysis

#### 3.6.1. Bioutilization and Bioavailability of Nutrients, Bioenergy Profiles and Carbohydrate Subfractions

Bioutilization and bioavailability of nutrients, bioenergy profiles and carbohydrate subfractions were analyzed using the PROC MIXED procedure of SAS 9.4 (SAS Institute, Inc., Cary, NC, USA) [26]. SAS model used for the analysis of was *Y*_ij_ = *u* + *trt*_i_ + *e*_ij_, where, *Y*_ij_ was the dependent variable; u was the mean for variable; *trt*_i_ was the effect of treatment (*i* = 1, 2, 3: alfalfa genetic types); *e*_ij_ was the random error. The non-transgenic (NT) and gene-silenced (GS) alfalfa populations were compared by Contrast statements in SAS 9.4 (SAS Institute, Inc., Cary, NC, USA). The two assumptions for CRD model were tested: (1) common variances among the three treatments and (2) residual data are normally distributed.

#### 3.6.2. Correlation Analysis of CHO Spectral Profiles with CHO Nutrient Supply

Correlations between CHO molecular structural profiles and bioutilization and bioavailability of nutrients, bioenergy profiles were analyzed using the PROC CORR procedure of SAS 9.4 (SAS Institute, Inc., Cary, NC, USA) [26] with a Spearman option after a normality test with Proc Univariate with Normal and Plot options. Shapiro-wilk was used to detect data normality in this study.

#### 3.6.3. Multiple Regression Analysis of CHO Spectral Profiles with Carbohydrate Subfractions, Bioenergy Value and Digestible Nutrients

Multiple regression analysis of CHO molecular structural profiles with CHO subfractions, bioenergy value and CHO nutrient digestion was performed to select the best IR parameters that explain nutrient values using the PROC REG procedure with a Stepwise option of SAS 9.4 (SAS Institute, Inc., Cary, NC, USA).

A Tukey–Kramer test was used to determine significant means among the treatments using pdmix 800 marco. Statistical significance was declared at *p* < 0.05 and trends were noted at 0.05 ≤ *p* ≤ 0.10.

## 4. Conclusions

In conclusion, RNAi-mediated silencing of *HB12* and *TT8* genes did not only affect inherent CHO molecular structure profiles but also affected their biofunctions. The structural CHO and non-structural CHO compounds in the alfalfa forage had different responses and different sensitivities to the silencing of *HB12* and *TT8* genes in the alfalfa forage. The gene silencing-induced CHO molecular structure changes were correlated with the biofunctions in terms of nutrient availability. 

## Figures and Tables

**Table 1:** Effect of *HB12* and *TT8* Down-regulation on Digestible Nutrient Profiles and Energy Values in Model Forage (*Medicago sativa* spp. *Sativa*: Alfalfa) ^a^.

Items	Non-Transgenic (NT)	Gene Silencing through RNAi Technology (GS)		SEM ^b^	*p* Value	Contrast, *p* Value
	Control	HB12	TT8			NT *vs.* GS
Truly digestible nutrient ^c^ (% DM)						
tdNFC	42.31 a	38.68 b	37.37 b	0.539	0.016	0.007
tdCPc	20.82 b	19.42 c	22.34 a	0.256	0.009	0.861
tdFA	0.57	0.88	0.45	0.131	0.197	0.605
tdNDF	14.49	14.78	14.89	0.622	0.898	0.679
Total digestible nutrient ^d^ (% DM)						
TDN1x	71.89	67.85	68.59	1.099	0.149	0.072
Predicted energy value ^e^ (Mcal/kg DM)						
DE_1x_	3.31	3.12	3.19	0.048	0.141	0.077
NE_m_	1.79	1.66	1.71	0.035	0.164	0.092
NE_g_	1.17	1.05	1.09	0.030	0.142	0.078
DE_3x_	3.04	2.86	2.93	0.044	0.143	0.081
ME_3x_	2.62	2.44	2.51	0.047	0.160	0.089
NE_LP_	1.65	1.52	1.57	0.031	0.140	0.075

^a^ means with different letters within the same row differ (*p* < 0.05); ^b^ SEM: stand error of mean; The Tukey-Kramer method was used for multi-treatmen comparison; ^c^ tdNFC: total digestible non-fiber carbohydrate; tdCPc: total digestible crude protein; tdFA: total digestible fatty acid; tdNDF: total digestible neutral detergent fiber; ^d^ TDN_1x_: total digestible nutrients at maintenance level; ^e^ DE_1x_: digestible energy at one times maintenance level; NE_m_: net energy for maintenance level; NE_g_: net energy for growth; DE_3x_: digestible energy at three times maintenance level; ME_3x_: metabolizable energy at three times maintenance level; NE_LP_: net energy for lactation at three times maintenance level.

**Table 2:** Effect of *HB12* and *TT8* down-regulation on carbohydrate subfractions in model forage (*Medicago sativa* spp. *Sativa*: Alfalfa) ^a^.

Items	Non-Transgenic (NT)	Gene silencing through RNAi Technology (GS)		SEM ^b^	*p* Value	Contrast, *p* Value
	Control	HB12	TT8			NT *vs.* GS
Fractions of Carbohydrate Partitioned by CNCPS ^c^ (% CHO)						
CA4	7.37	4.18	4.41	0.867	0.135	0.063
CB1	5.26 ^a^	2.04 ^b^	2.58 ^b^	0.334	0.012	0.006
CB2	49.35	49.79	49.41	1.201	0.962	0.876
CB3	29.89	29.64	31.30	1.534	0.735	0.779
CC	8.14	14.36	12.31	1.908	0.209	0.113

^a^ means with different letters within the same row differ significantly *(p* < 0.05); ^b^ SEM: stand error of the mean; The Tukey-Kramer method was used for multi-treatment comparison; ^c^ CNCPS: Cornell Net Carbohydrate and Protein System; Carbohydrate CNCPS fractions undetected in alfalfa populations include: CA1 fraction (volatile fatty acids), CA2 fraction (lactate acid), CA3 (other organic acids). Measurable CNCPS alfalfa carbohydrate fractions include: CA4 (sugar), CB1 (starch), CB2 (soluble fiber), CB3 (available NDF) and CC (indigestible fiber).

**Table 3:** Correlation between carbohydrate-related molecular spectral characteristics and truly digestible nutrients in model forage (*Medicago sativa* spp*. Sativa*: Alfalfa).

Item ^a^	TDN1x % DM		tdNFC % DM		tdFA % DM		tdNDF % DM	
	*r* ^b^	*p* Value	*r*	*p* Value	*r*	*p* Value	*r*	*p* Value
Structural CHO related Spectral Profiles								
A_StCHO	−0.09	0.872	0.09	0.872	−0.54	0.266	−0.81	0.050
H_1415	0.19	0.725	0.37	0.470	−0.49	0.320	−0.70	0.118
H_1370	−0.06	0.913	0.12	0.827	−0.61	0.200	−0.87	0.025
H_1315	0.00	1.000	0.09	0.868	−0.71	0.117	−0.85	0.032
A_1315	−0.83	0.042	−0.83	0.042	−0.14	0.787	−0.32	0.538
Cellulosic Compounds Related Spectral Profiles								
H_1244	0.09	0.868	0.00	1.000	−0.85	0.031	−0.72	0.109
A_CELC	0.09	0.872	−0.09	0.872	−0.83	0.042	−0.55	0.257
Total CHO related Spectral Profiles								
A_CHO	0.03	0.957	0.03	0.957	−0.71	0.111	−0.75	0.084
H_1150	−0.03	0.957	0.06	0.913	−0.64	0.173	−0.79	0.059
H_1100	−0.03	0.957	0.06	0.913	−0.64	0.173	−0.79	0.059
H_1025	−0.03	0.957	0.06	0.913	−0.64	0.173	−0.79	0.059
Non-structural CHO related Spectral Profiles								
A_non-st CHO	−0.09	0.872	0.09	0.872	−0.54	0.266	−0.81	0.500
H_895	−0.13	0.805	0.39	0.441	0.13	0.805	−0.66	0.150
Spectral Ratio Profiles								
A_StCHO/A_CHO ratio	0.49	0.329	0.71	0.111	−0.09	0.872	−0.26	0.618
A_Non-stCHO/A_CHO ratio	−0.41	0.414	0.00	1.000	0.83	0.042	0.11	0.843
A_Non-stCHO/A_StCHO ratio	−0.21	0.695	0.03	0.956	0.91	0.011	0.40	0.428
A_CELC/A_CHO ratio	−0.03	0.957	−0.43	0.397	−0.94	0.005	−0.32	0.538
A_CELC/A_StCHO ratio	−0.09	0.872	−0.54	0.266	−0.54	0.266	0.06	0.913

^a^ r: correlation coefficient; ^b^ TDN_1x_: total digestible nutrient at maintenance level; tdNFC: total digestible non-fiber carbohydrate; tdFA: total digestible fatty acid; tdNDF: total digestible neutral detergent fiber.

**Table 4:** Correlation between carbohydrate molecular spectral characteristics and carbohydrate cncps fractions in model forage (*Medicago sativa* spp. *Sativa*: Alfalfa).

Item ^a^	CA4 % CHO		CB1 % CHO		CB2 % CHO		CB3 % CHO		CC % CHO	
	*r* ^b^	*p* Value	*r*	*p* Value	*r*	*p* Value	*r*	*p* Value	*r*	*p* Value
Structural CHO related Spectral Profiles										
A_StCHO	0.71	0.111	0.71	0.111	−0.60	0.208	−0.77	0.072	0.09	0.872
H_1415	0.83	0.039	0.83	0.039	−0.62	0.192	−0.62	0.192	−0.19	0.725
H_1370	0.75	0.084	0.75	0.084	−0.70	0.125	−0.81	0.050	0.06	0.913
H_1315	0.79	0.059	0.79	0.059	−0.62	0.191	−0.79	0.059	0.00	1.000
A_1315	−0.43	0.397	−0.43	0.397	−0.14	0.787	−0.43	0.397	0.83	0.042
Cellulosic Compounds Related Spectral Profiles										
H_1244	0.79	0.059	0.79	0.059	−0.47	0.346	−0.65	0.165	−0.09	0.868
A_CELC	0.71	0.111	0.71	0.111	−0.31	0.544	−0.49	0.329	−0.09	0.872
Total CHO related Spectral Profiles										
A_CHO	0.77	0.072	0.77	0.072	−0.43	0.397	−0.71	0.111	-0.03	0.957
H_1150	0.75	0.084	0.75	0.084	−0.52	0.288	−0.75	0.084	0.03	0.957
H_1100	0.75	0.084	0.75	0.084	−0.52	0.288	−0.75	0.084	0.03	0.957
H_1025	0.75	0.084	0.75	0.084	−0.52	0.288	−0.75	0.084	0.03	0.957
Non-structural CHO related Spectral Profiles										
A_non-st CHO	0.71	0.111	0.71	0.111	-0.60	0.208	−0.77	0.072	0.09	0.872
H_895	0.39	0.441	0.39	0.441	-0.65	0.158	−0.65	0.158	0.13	0.805
Spectral Ratio Profiles										
A_StCHO/A_CHO ratio	0.66	0.156	0.66	0.156	−0.43	0.397	−0.14	0.787	−0.49	0.329
A_Non-stCHO/A_CHO ratio	−0.62	0.188	−0.62	0.188	0.00	1.000	0.00	1.000	0.41	0.414
A_Non-stCHO/A_StCHO ratio	−0.65	0.165	−0.65	0.165	0.50	0.312	0.26	0.612	0.21	0.695
A_CELC/A_CHO ratio	0.37	0.469	0.37	0.469	−0.20	0.704	−0.26	0.623	0.03	0.957
A_CELC/A_StCHO ratio	0.03	0.957	0.03	0.957	0.37	0.469	0.03	0.957	0.09	0.872

^a^ CNCPS: Cornell Net Carbohydrate and Protein System; Carbohydrate CNCPS fractions undetected in alfalfa populations include: CA1 fraction (volatile fatty acids), CA2 fracton (lactic acid), and CA3 fraction (other organic acids). Measurable CNCPS alfalfa carbohydrate fractions include: CA4 (sugar), CB1 (starch), CB2 (soluble fiber), CB3 (available NDF) and CC (indigestible fiber); ^b^ *r*: correlation coefficient.

**Table 5:** Multiple regression analysis to choose the most important CHO spectral parameters to predict CHO nutrient supply from the alfalfa forage (*Medicago sativa* spp. *Sativa*).

Predicted Variables (*Y*)	Variable Selection (Variables Left in the Model with *p* < 0.05)	Prediction Equations (Test Model: *Y* = *a* + *b*_1_*x*_1_ + *b*_2_*x*_2_)	Model *R*^2^ Value	RSD ^a^	*p* Value
Carbohydrate fraction profiles					
CA4 (% CHO)	Ratio of structural CHO to total CHO left in the model	*Y* = −41.04 + 99.32 A_StCHO/A_CHO ratio	0.77	1.00	0.022
CB1 (% CHO)	H_1415 left in the model	*Y* = −6.91 + 355.77 H_1415	0.70	0.97	0.038
CB3 (% CHO)	H_895 left in the model	*Y* = 42.57 − 4338.00 H_895	0.91	0.64	0.004
Total digestible nutrients					
tdNDF (% DM)	A_non-STCHO left in the model	*Y* = 21.84 − 83.78 A_non-STCHO	0.88	0.28	0.006

^a^ RSD: Residual standard deviation.
